# Dataset on noise level measurement in Ota metropolis, Nigeria

**DOI:** 10.1016/j.dib.2018.12.049

**Published:** 2018-12-19

**Authors:** S.O. Oyedepo, G.A. Adeyemi, O.S.I. Fayomi, O.K. Fagbemi, R. Solomon, T. Adekeye, O.P. Babalola, M.L. Akinyemi, O.C. Olawole, E.S. Joel, S.C. Nwanya

**Affiliations:** a Department of Mechanical Engineering, Covenant University, Nigeria; b Department of Civil Engineering, Covenant University, Nigeria; c Department of Physics, Covenant University, Nigeria; d Department of Mechanical Engineering, University of Nigeria, Nsukka, Nigeria

**Keywords:** Noise pollution, Noise descriptors, Traffic noise, Road junctions, Noise map, Peak noise level, Background noise level

## Abstract

Datasets contained in this article are noise level measurement carried out at 41 different locations in Ota metropolis, Nigeria. The noise readings were measured at a time interval of 30 min for each site considered using a precision grade sound level meter. The analysis was based on the noise descriptors L_Aeq_, L_10_, L_90_, L_D_, TNI and NEI. Results from the study reflects that the highest and lowest equivalent noise levels (L_Aeq_) were recorded at commercial areas (96 dB (A)) and residential areas (52 dB (A)), respectively, the background noise level (L_90_) has the highest and lowest values at commercial areas (77 dB (A)) and residential areas (44 dB (A)), respectively and the peak value (L_10_) has the highest value and lowest value at the commercial areas (96 dB (A)) and residential areas (56 dB (A)). Based on the WHO recommendations and standards, only 2 out of the 41 locations considered are under normally acceptable situation while the noise levels of other areas are not acceptable. Noise map developed in this study provides enough information for technical controls and interim legislation against environmental noise pollution in the metropolis. Moreover, considering the noise emission standards, planning and promoting the citizens awareness about the high noise risk could help to mitigate the effect of noise in Ota, Metropolis. The noise data in this study are useful as reference and guideline for future regulations on noise limit to be implemented for urban areas in Nigeria and developing countries at large.

**Specifications table**

**Table:** 

Subject Area	Engineering
More Specific Subject area	Mechanical Engineering, Environmental Engineering, Environmental Noise Control
Type of Data	Tables, Figures and graphs
How Data was Acquired	Field noise readings was carried out at 41 – selected locations using Sound Level Meter (SLM) (model: 8922 Digital Sound Level Meter).
Data Format	Raw and analysed
Experimental Factor	Noise level at a total number of 41 locations comprises of commercial areas, industrial areas, busy roads & road junctions, passengers loading parks and residential areas in Ota Metropolis was carried out.
Experimental Feature	Instrumentation for the field measurements consisted of precision grade sound level meter (according to IEC 651, ANSI S1.4 type 2 class standards), 1/2-in. condenser microphone and 1/3-octave filter with frequency range and measuring level range of 31.5 Hz–8 KHz and 30–130 dB respectively. The instruments were calibrated by the internal sound level calibrator before making measurements at each site. All the instruments comply with IEC standards. Details procedures for the environmental noise measurements can be found in Refs. [1], [2], [3]. *L*_Ai_ (A-weighted instantaneous Sound pressure level) measurements were recorded at intervals of 30 s for a period of 30 min, giving 60 m readings per sampling location. This procedure was carried out for morning (7:00–9:00 a.m.), afternoon (1:00–3:00 p.m.) and evening (6:00–8:00 p.m.) measurements. From these readings, commonly used community noise descriptors such as minimum noise level (*L*_min_), maximum noise level (*L*_max_), equivalent A-weighted sound pressure level (*L*_Aeq_), Noise Pollution Level (LNP), Traffic Noise Index (TNI), Noise Climate (NC), Noise Emission Index (NEI), Day Noise Levels (*L*_D_), Night Noise Levels (*L*_N_), the exceedence percentiles (*L*_10_, *L*_50_, *L*_90_), Noise climate (NC) and Traffic noise index (TNI) were computed.
Data source location	Ota Metropolis, Nigeria
Data Accessibility	Data are available within this article

**Value of the data**

• The given data can be used to develop noise map for Urban Cities in Nigeria and other developing countries.
• The data contained herein can help to establish environmental noise impact criteria levels for various land use purposes. These criteria levels would enable impacts to be determined.
• The given data will show researchers in the field of environmental management and sustainable city development the trend of noise pollution as it relate to commercial activities, industrial activities and traffic volume in urban areas.
• The data on noise level measurement can be used in creation of a database for urban planning with localisation of noisy activities and mixed and sensitive zones.
• The data can be used to evaluate population exposure to noise pollution in urban area.

## 1 Data

**Fig. 1:**
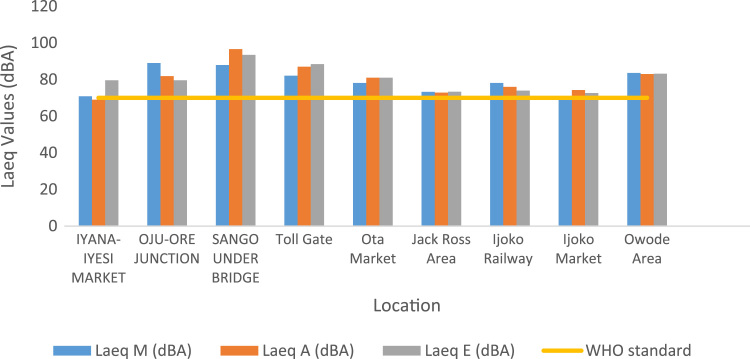
Variation of equivalent noise levels in commercial areas and comparison with WHO standards.

**Fig. 2:**
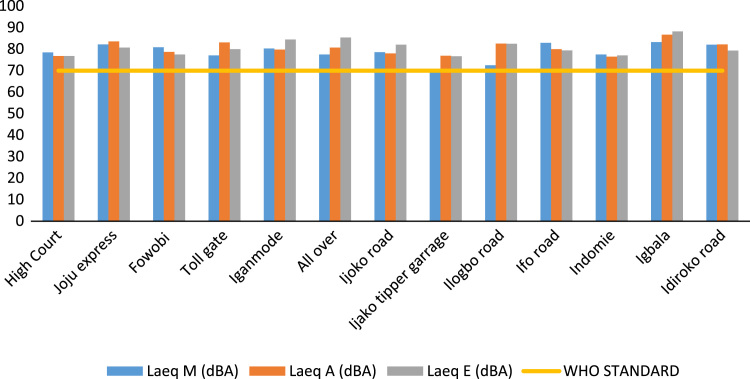
Variation of equivalent noise levels at major roads and comparison with WHO standards.

**Fig. 3:**
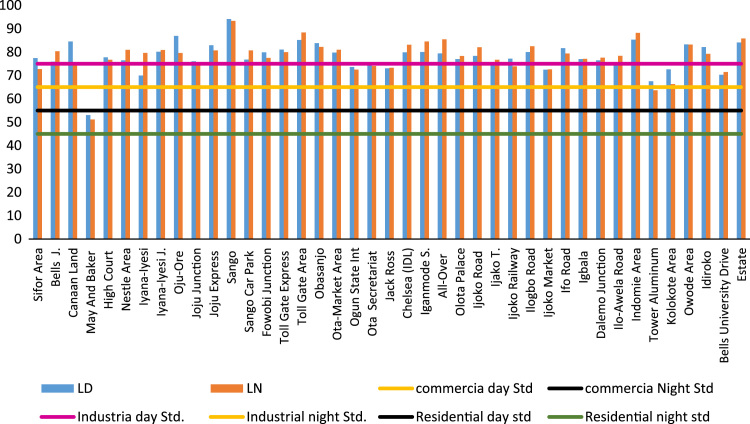
Variation of *L*_D_ and *L*_N_ values in the selected locations.

To assess the noise pollution levels in Ota Metropolis, 41 locations were selected for study. These locations were grouped into commercial area, industrial areas, road junctions/busy roads and residential areas. The noise descriptors for each location at respective time of the day are presented in Table 1, Table 2, Table 3. Figs. 1 and 2 show the variations and comparison of equivalent noise level at commercial areas and major roads with WHO standards. Fig. 3 presents variations of L_D_ and L_N_ values in the selected locations.

**Table 1:** Minimum (*L*_min_), Maximum (*L*_max_) and percentile noise exceeded (*L*_10_, *L*_50_, *L*_90_) at selected locations.

Location	*L*_min_ dB(A)			*L*_max_ dB(A)			*L*_10_ dB(A)			*L*_50 dB_(A)			*L*_90_ dB(A)		
	M	A	E	M	A	E	M	A	E	M	A	E	M	A	E
Sifor Area	66	65	61	82	86	82	72	88	76	71	75	70	66	68	63
Bells University Junction	63	66	69	78	89	92	77	84	80	73	74	75	69	69	72
Canaan Land	65	65	63	90	101	85	80	88	78	72	75	72	67	69	67
May And Baker Close	45	43	31	67	65	63	56	56	55	48	49	45	46	45	41
High Court Area	63	63	65	86	89	83	82	79	80	77	75	76	72	70	70
Nestle	68	64	66	88	82	97	80	78	80	75	74	74	72	68	69
Iyana-Iyesi Market	59	60	77	81	78	87	74	72	82	66	67	79	63	62	78
Iyana-Iyesi Junction	69	69	72	95	86	89	84	83	85	78	75	78	73	71	74
Oju-Ore Junction	75	71	71	105	93	87	90	85	83	81	76	78	77	73	74
Joju Junction	63	63	67	89	84	84	79	79	79	74	73	73	68	69	69
Joju Express Road	73	72	73	88	94	94	86	88	82	80	80	77	75	75	75
Sango Under Bridge	73	75	73	102	113	110	91	96	87	79	83	78	75	77	75
Sango Car Park	60	55	67	92	89	94	80	80	84	68	71	73	61	71	68
Fowobi Junction	67	70	67	93	87	87	84	84	80	76	76	76	71	72	72
Toll Gate Express	67	65	71	85	98	88	86	76	85	74	71	78	70	67	73
Toll Gate Area	70	70	73	91	103	99	86	86	92	78	77	84	73	72	76
Obasanjo Junction	68	70	70	92	93	91	85	90	85	76	80	79	71	72	74
Ota-Market Area	66	68	67	85	94	94	82	84	83	75	77	77	70	72	71
Ogun State Internal Revenue	58	60	64	89	81	85	77	74	75	67	67	69	60	62	65
Ota Local Government Sct	63	63	63	91	87	82	77	75	77	71	70	72	66	65	67
Jack Ross Area (Road)	57	52	59	86	84	82	76	73	79	69	66	70	62	67	64
Chelsea (IDL)	55	57	69	99	83	90	80	78	87	72	71	82	63	63	75
Iganmode Sec School A/R	67	65	65	91	90	92	83	84	90	76	76	90	72	72	74
All-Over Polytechnic Road	61	64	75	84	95	94	82	84	90	76	74	81	65	66	76
Olota Palace Junction	66	65	69	90	86	86	80	78	80	74	73	78	70	68	73
Ijoko Road	66	59	53	90	84	95	82	81	85	74	77	79	70	68	71
Ijako Tipper Garrage	60	60	60	82	88	89	73	83	78	67	70	72	62	65	67
Ijoko Railway Station	59	64	53	91	82	81	81	80	78	70	75	72	62	69	65
Ilogbo Road	61	64	63	82	90	91	76	87	86	70	79	80	66	68	74
Ijoko Market	57	58	58	77	80	79	75	78	78	67	73	70	61	67	62
Ifo Road	68	66	68	93	88	87	86	83	83	80	79	78	71	72	70
Owode Area	64	65	56	88	82	85	80	80	80	74	78	76	68	68	66
Dalemo Junction	65	64	63	82	86	84	78	82	81	72	76	76	68	68	69
Ilo-Awela Road	60	62	66	84	83	84	74	81	82	68	73	77	63	66	71
Indomie	71	73	68	94	97	99	87	91	94	80	83	81	75	76	73
Tower Aluminum Company	51	50	48	79	72	71	75	71	68	59	64	59	55	55	53
Kolokote Area	55	51	52	87	73	74	81	61	70	62	59	63	56	54	56
Owode Area	64	69	64	92	91	90	89	86	88	78	80	79	73	75	73
Idiroko Road (Chelsea Area)	61	67	65	92	89	87	86	87	83	78	81	77	70	74	70
Bells University Drive	49	52	50	84	76	80	76	73	76	63	67	69	54	58	56
Estate	55	68	65	96	93	97	90	88	90	78	79	78	70	74	69

Key: M – Morning; A – Afternoon; E – Evening.

**Table 2:** Traffic noise Index (TNI), Pollution noise level (LNP) and Average Equivalent Noise Levels (*L*_Aeq_) for the selected locations.

Location	TNI dB(A)			LNP dB(A)			*L*_Aeq_ dB(A)		
	M	A	E	M	A	E	M	A	E
Sifor Area	60	118	85	79.29	99.55	85.74	73.29	79.55	72.74
Bells University Junction	71	99	74	81.65	92.35	88.33	73.65	77.35	80.33
Canaan Land	89	115	81	90.65	106.09	86.06	77.65	87.09	75.06
May And Baker Close	56	59	67	62.68	64.42	65.14	52.68	53.42	51.14
High Court Area	82	76	80	88.52	85.82	86.71	78.52	76.82	76.71
Nestle Area	74	78	83	85.36	85.32	91.95	77.36	75.32	80.95
Iyana-Iyesi Market	77	72	64	81.83	78.93	83.63	70.83	68.93	79.63
Iyana-Iyesi Junction	87	89	88	92.44	90.18	91.89	81.44	78.18	80.89
Oju-Ore Junction	99	91	80	102.18	93.80	88.59	89.18	81.80	79.59
Joju Junction	82	79	79	87.79	85.20	85.13	76.79	75.20	75.13
Joju Express Road	89	97	73	93.16	96.62	87.73	82.16	83.62	80.73
Sango Under Bridge	109	123	93	103.92	115.57	105.36	87.92	96.57	93.36
Sango Car Park	107	77	102	95.82	85.80	96.73	76.82	76.80	80.73
Fowobi Junction	93	90	74	93.88	90.69	85.48	80.88	78.69	77.48
Toll Gate Express	104	73	91	93.14	92.11	91.95	77.14	83.11	79.95
Toll Gate Area	95	98	110	95.10	100.98	104.39	82.10	86.98	88.39
Obasanjo Junction	97	114	88	94.68	103.59	93.20	80.68	85.59	82.20
Ota-Market Area	88	90	89	90.01	93.03	92.98	78.01	81.03	80.98
Ogun State Internal Revenue Area	98	80	75	92.51	82.01	82.50	75.51	70.01	72.50
Ota Local Government Secretariat	80	75	77	87.10	83.30	84.04	76.10	73.30	74.04
Jack Ross Area (Road)	88	61	94	87.28	78.78	88.31	73.28	72.78	73.31
Chelsea (IDL)	101	93	93	99.43	88.22	95.15	82.43	73.22	83.15
Iganmode Sec School Area/Road	86	90	108	91.27	91.82	100.52	80.27	79.82	84.52
All-Over Polytechnic Road	103	108	102	94.53	98.73	99.43	77.53	80.73	85.43
Olota Palace Junction	80	78	71	88.04	85.59	85.30	78.04	75.59	78.30
Ijoko Road	88	90	97	90.64	91.03	96.06	78.64	78.03	82.06
Ijako Tipper Garrage	76	107	81	81.13	94.91	87.72	70.13	76.91	76.72
Ijoko Railway Station	108	83	87	97.05	87.12	86.89	78.05	76.12	73.89
Ilogbo Road	76	114	92	82.45	101.60	94.49	72.45	82.60	82.49
Ijoko Market	87	81	96	83.70	85.18	88.62	69.70	74.18	72.62
Ifo Road	101	86	92	97.85	91.01	92.40	82.85	80.01	79.40
Igbala	86	86	92	89.48	88.48	91.11	77.48	76.48	77.11
Dalemo Junction	78	94	87	84.22	91.93	89.62	74.22	77.93	77.62
Ilo-Awela Road	77	96	85	83.01	91.64	89.41	72.01	76.64	78.41
Indomie Area	93	106	127	95.34	101.67	109.22	83.34	86.67	88.22
Tower Aluminum Company	105	89	83	88.59	82.05	78.66	68.59	66.05	63.66
Kolokote Area	126	52	82	100.30	71.10	80.29	75.30	64.10	66.29
Owode Area	107	89	103	99.61	93.88	98.21	83.61	82.88	83.21
Idiroko Road(Chelsea Area)	104	96	92	98.07	95.21	92.26	82.07	82.21	79.26
Bells Drive	112	88	106	93.56	83.48	91.48	71.56	68.48	71.48
Estate	120	100	123	104.57	97.62	106.85	84.57	83.62	85.85

Key: M – Morning; A – Afternoon; E – Evening.

**Table 3:** Noise Climate (NC) and Noise Exposure Index (NEI) for the selected locations.

**Location**	**NEI**			**Noise climate**			***L***_**Day**_	***L***_**Night**_
	**M**	**A**	**E**	**M**	**A**	**E**	**(*L***_**D**_**)**	**(*L***_**N**_**)**
Sifor Area	1.04	1.14	1.04	6	20	13	77.5	72.7
Bells University Junction	1.05	1.11	1.15	8	15	8	75.9	80.3
Canaan Land	1.11	1.24	1.07	13	19	11	84.6	75.1
May And Baker Close	0.96	0.97	0.93	10	11	14	53.1	51.1
High Court Area	1.12	1.12	1.10	10	9	10	77.6	76.7
Nestle Area	1.05	1.08	1.16	8	10	11	76.5	81.0
Iyana-Iyesi Market	1.09	1.06	1.45	11	10	4	70.0	79.6
Iyana-Iyesi Junction	1.16	1.12	1.16	11	12	11	80.1	80.9
Oju-Ore Junction	1.37	1.26	1.45	13	12	9	86.9	79.6
Joju Junction	1.10	1.07	1.07	11	10	10	76.1	75.1
Joju Express Road	1.17	1.20	1.11	11	13	7	83.0	80.7
Sango Under Bridge	1.35	1.49	1.70	16	19	12	94.1	93.4
Sango Car Park	1.10	1.10	1.15	19	9	16	76.8	80.7
Fowobi Junction	1.16	1.12	1.11	13	12	8	79.9	77.5
Toll Gate Express	1.10	1.19	1.14	16	9	12	81.1	80.0
Toll Gate Area	1.26	1.34	1.61	13	14	16	85.2	88.4
Obasanjo Junction	1.15	1.22	1.17	14	18	11	83.8	82.2
Ota-Market Area	1.20	1.25	1.47	12	12	12	79.8	81.0
Ogun State Internal Revenue Area	1.37	1.27	1.61	17	12	10	73.6	72.5
Ota Local Government Sect	1.38	1.33	1.65	11	10	10	74.9	74.0
Jack Ross Area (Road)	1.09	1.06	1.45	14	6	15	73.0	73.3
Chelsea (IDL)	1.10	0.98	1.28	17	15	12	79.9	83.2
Iganmode Sec School A/R	1.15	1.14	1.21	11	12	16	80.1	84.5
All-Over Polytechnic Road	1.11	1.14	1.22	17	18	14	79.4	85.4
Olota Palace Junction	1.20	1.16	1.42	10	10	7	77.0	78.3
Ijoko Road	1.12	1.11	1.17	12	13	14	78.4	82.1
Ijako Tipper Garrage	1.00	1.10	1.10	11	18	11	74.7	76.7
Ijoko Railway Station	1.20	1.17	1.34	19	11	13	77.2	73.9
Ilogbo Road	1.04	1.18	1.19	10	19	12	80.0	82.5
Ijoko Market	1.07	1.14	1.32	14	11	16	72.5	72.6
Ifo Road	1.18	1.14	1.13	15	11	13	81.7	79.4
Igbala	1.11	1.10	1.10	12	12	14	77.0	77.1
Dalemo Junction	1.06	1.11	1.11	10	14	12	76.5	77.6
Ilo-Awela Road	1.03	1.10	1.12	11	15	11	74.9	78.4
Indomie Area	1.19	1.24	1.26	12	15	21	85.3	88.2
Tower Aluminum Company	0.91	0.88	0.98	20	16	15	67.5	63.7
Kolokote Area	1.00	0.85	1.02	25	7	14	72.6	66.3
Owode Area	1.29	1.28	1.51	16	11	15	83.3	83.2
Idiroko Road(Chelsea Area)	1.17	1.17	1.13	16	13	13	82.1	79.3
Bells University Drive	1.10	1.05	1.30	22	15	20	70.3	71.5
Estate	1.13	1.11	1.32	20	14	21	84.1	85.9

## 2 Experimental design, materials and methods

The noise measurements were made at the street level (at road junctions/busy roads, commercial centres, industrial areas and residential areas). The instrument (sound level meter) used was held comfortably in hand with the microphone pointed at the suspected noise source at a distance not less than 1 m away from any reflecting object. *L*_Ai_ (A-weighted instantaneous sound pressure level) measurements were recorded at intervals of 30 s for a period of 30 min, giving 60 readings per sampling location. This procedure was carried out for morning (7:00–8:00 a.m.), afternoon (1:00–3:00 p.m.) and evening (6:00–8:00 p.m.) measurements. From these readings, commonly used community noise assessment quantities such as minimum noise level (*L*_min_), maximum noise level (*L*_max_), equivalent A-weighted sound pressure level (*L*_Aeq_), Noise Pollution Level (LNP), Traffic Noise Index (TNI), Noise Climate (NC), Noise Emission Index (NEI), Day Noise Levels (LD), Night Noise Levels (LN), the exceedence percentiles (*L*_10_, *L*_50_, *L*_90_), Noise climate (NC) and Traffic noise index (TNI) were computed.

According to the Directive 2002/49/EC of the European Parliament and of the Council, of 25 June 2002 relating to the assessment and management of environmental noise imposes to its Member States the elaboration of noise maps for cities with more than 250,000 inhabitants, this was due on 30 June 2007 [4], [5]. Based on this directive, Ota metropolis with population of over 527,242 inhabitants is qualified to be presented with noise map.

**Fig. 4:**
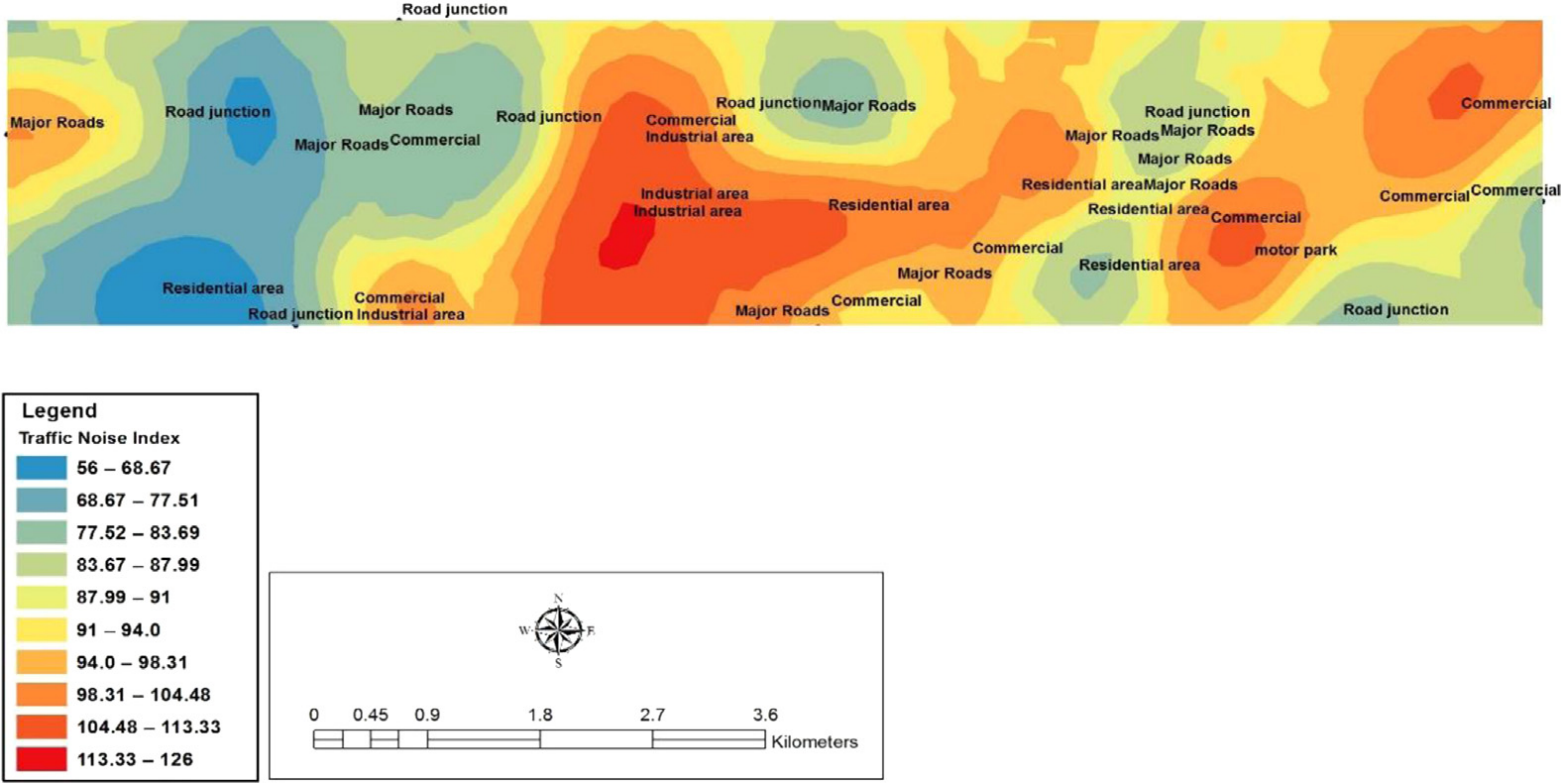
Spatial variation mapping of noise levels in Ota metropolis for the morning period.

**Fig. 5:**
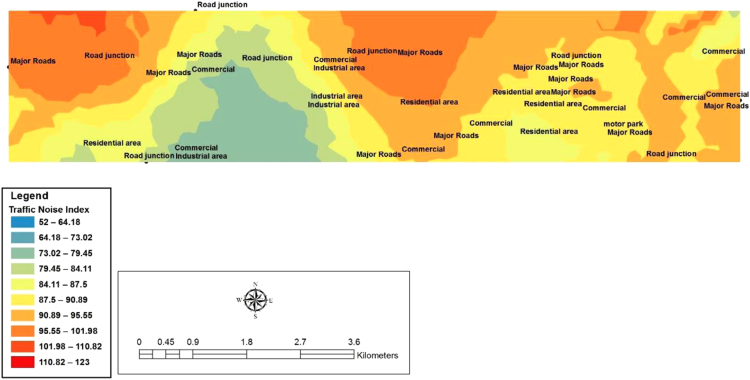
Spatial variation mapping of noise levels in Ota metropolis for the afternoon period.

**Fig. 6:**
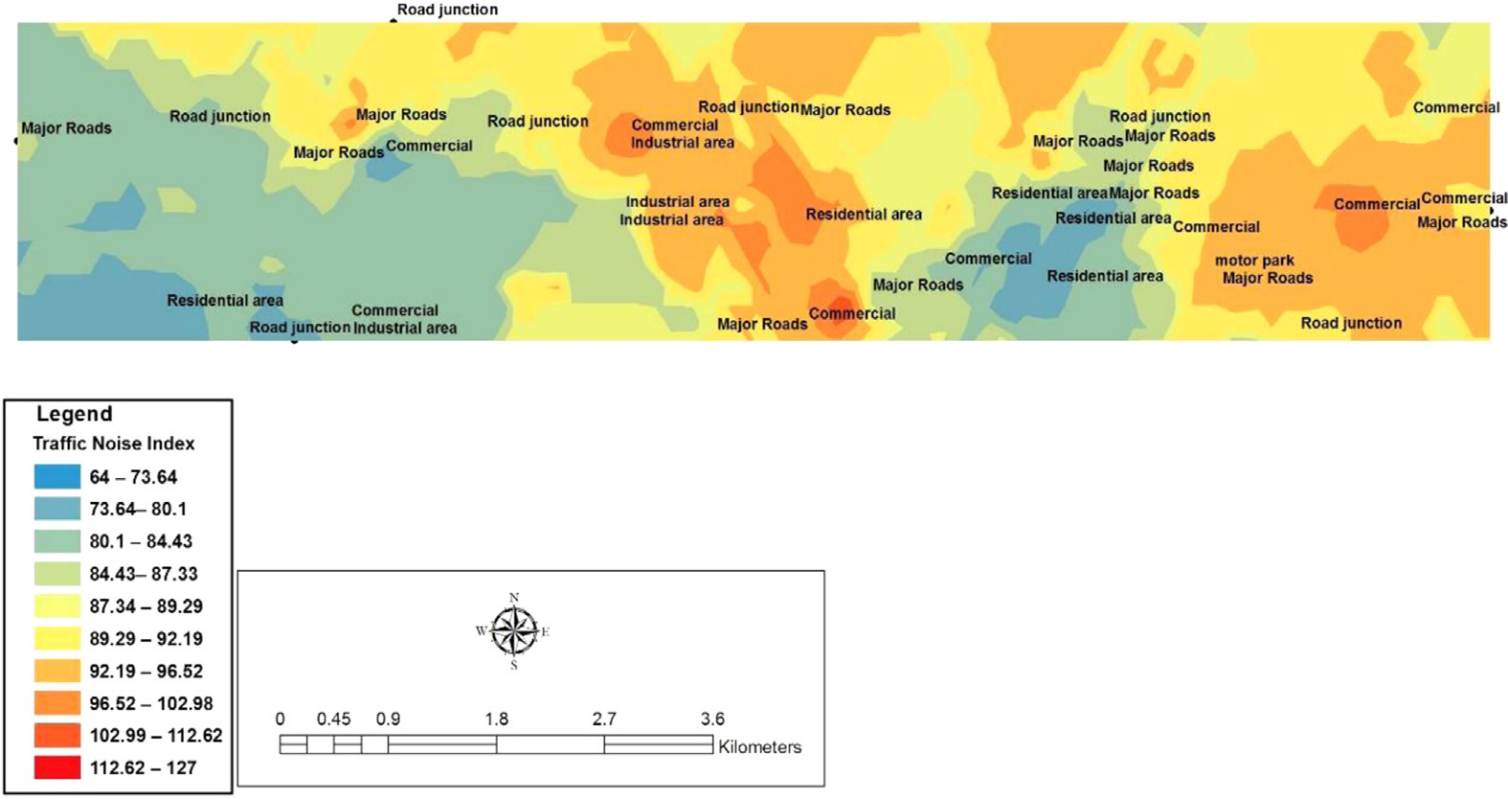
Spatial variation mapping of noise levels in Ota metropolis in the evening.

In this study, ArcGIS 10.5 Software was used to develop the spatial variability mapping of Ota with the use of Inverse Diverse Weighting (IDW) interpolation method. The codes adopted and the geographical positioning systems Coordinates for the 41 chosen locations surveyed in Ota metropolis are shown below in Table 4. Fig. 4, Fig. 5, Fig. 6 show the spatial variation mapping of noise levels in Ota metropolis for the morning, afternoon and evening periods of the day.

**Table 4:** Geographical positioning systems coordinates for the selected locations.

**S/N**	**Location**	**Latitude**	**Longitude**	**Elevation (m)**
1	Sifor Area	6° 40′ 57.8′′	3° 10′ 24.3′′	75
2	Bells University Junction	6° 41′ 00.2′′	3° 10′ 38.2′′	63
3	Canaan Land	6° 40′ 55.7′′	3° 10′ 03.7′′	63
4	May And Baker Close	6° 41′ 07.2′′	3° 10′ 03.1′′	62
5	High Court Area	6° 40′ 52.7′′	3° 11′ 02.7′′	55
6	Nestle Area	6° 40′ 54.4′′	3° 11′ 29.2′′	52
7	Iyana-Iyesi Market	6° 40′ 48.0′′	3° 11′ 01.8′′	64
8	Iyana-Iyesi Junction	6° 40′ 83.9′′	3° 11′ 04.9′′	65
9	Oju-Ore	6° 41′ 18.2′′	3° 13′ 32.3′′	73
10	Joju Junction	6° 41′ 55.8′′	3° 14′ 16.7′′	77
11	Joju Express Road	6° 42′ 35.6′′	3° 14′ 16.5′′	78
12	Sango Under Bridge	6° 42′ 26.6′′	3° 14′ 33.7′′	85
13	Sango Car Park	6° 42′ 17.8′′	3° 14′ 45.2′′	82
14	Fowobi Junction	6° 41′ 11.3′′	3° 13′ 13.0′′	81
15	Toll Gate Express	6° 42′ 19.3′′	3° 14′ 47.2′′	80
16	Toll Gate Area	6° 41′ 32.6′′	3° 15′ 25.6′′	80
17	Obasanjo Junction	6° 40′ 58.2′′	3° 12′ 35.0′′	68
18	Ota-Market Area	6° 41′ 03.8′′	3° 12′ 55.7′′	68
19	Ogun State Internal Revenue Area	6° 41′ 35.6′′	3° 14′ 12.2′′	80
20	Ota Local Government Secretariat	6° 41′ 29.0′′	3° 14′ 12.1′′	72
21	Jack Ross Area (Road)	6° 40′ 04.6′′	3° 10′ 52.6′′	53
22	Chelsea (IDL)	6° 40′ 04.4′′	3° 10′ 53.2′′	67
23	Iganmode Sec School Area/Road	6° 40′ 56.3′′	3° 10′ 53.8′′	88
24	All-Over Polytechnic Road	6° 41′ 49.2′′	3° 13′ 59.8′′	84
25	Olota Palace Junction	6° 41′ 13.6′′	3° 13′ 59.9′′	78
26	Ijoko Road	6° 40′ 57.4′′	3° 12′ 30.7′′	68
27	Ijako Tipper Garrage	6° 44′ 34.3′′	3° 15′ 59.9′′	90
28	Ijoko Railway Station	6° 44′ 58.0′′	3° 15′ 38.4′′	71
29	Ilogbo Road	6° 44′ 57.4′′	3° 12′ 53.4′′	46
30	Ijoko Market	6° 44′ 34.2′′	3° 15′ 60.0′′	92
31	Ifo Road	6° 45′ 00.9′′	3° 12′ 53.0′′	47
32	Igbala	6° 42′ 42.7′′	3° 13′ 75.0′′	69
33	Dalemo Junction	6° 42′ 01.3′′	3° 15′ 08.1′′	60
34	Ilo-Awela Road	6° 41′ 50.7′′	3° 14′ 20.9′′	83
35	Indomie Area	6° 41′ 08.0′′	3° 13′ 05.9′′	78
36	Tower Aluminum Company	6° 40′ 33.2′′	3° 12′ 06.5′′	67
37	Kolokote Area	6° 40′ 28.5′′	3° 12′ 04.8′′	74
38	Owode Area	6° 40′ 53.4′′	3° 12′ 07.9′′	69
39	Idiroko Road(Chelsea Area)	6° 40′ 52.7′′	3° 09′ 23.7′′	64
40	Bells Drive	6° 44′ 30.0′′	3° 12′ 55.0′′	51
41	Estate	6° 40′ 53.4′′	3° 12′ 07.9′′	71

According to recommendation of noise levels for specific environments by WHO 2002 (in Arokoyu et al. [6] and Usikalu and Kolawole [7]), all the locations surveyed in this study are categorized based on the noise maps developed into three different zones which are (a) low risk zone (May and baker location) (b) Moderate risk zone (Iyana-Iyesi market, Ilogbo road area, Tower aluminium area, Bells University drive) and (c) High risk zone (Sango under bridge, Oju Ore, Canaan Land area, Nestle area, Idiroko road, Estate and other locations with L_Aeq_ exceeds 81 dB (A)).
