# Supplementary material for: Dataset on noise level measurement in Ota metropolis, Nigeria
Source: Data Brief. 2018 Dec 19;22:762–70. doi: 10.1016/j.dib.2018.12.049 (PMC6330360; doi:10.1016/j.dib.2018.12.049)
Supplement: Supplementary file 1 — Supplementary material [file mmc1.docx]

**CONFLICT OF INTEREST**

On behalf of other co-authors of this paper, I hereby declare that there is no conflict of interest as this manuscript being submitted to Data in Brief.

Kind regards

Oyedepo, S.O
